# Perspective: The Impact of Fasting and Caloric Restriction on Neurodegenerative Diseases in Humans

**DOI:** 10.1016/j.advnut.2024.100197

**Published:** 2024-03-01

**Authors:** Bérénice Hansen, Kirsten Roomp, Hebah Ebid, Jochen G Schneider

**Affiliations:** 1 Luxembourg Centre for Systems Biomedicine, University of Luxembourg, Esch-sur-Alzette, Luxembourg; 2 Departments of Internal Medicine II and Psychiatry, Saarland University Medical Center, Homburg, Germany

**Keywords:** neurodegenerative disease, fasting, caloric restriction, ketogenic diet, Alzheimer’s disease, Parkinson’s disease, multiple sclerosis, mild cognitive impairment, elderly, human

## Abstract

Neurodegenerative diseases (NDs) are characterized by the progressive functional and structural denaturation of neurons in the central and peripheral nervous systems. Despite the wide range of genetic predispositions, the increased emergence of these disorders has been associated with a variety of modifiable risk factors, including lifestyle factors. Diet has been shown to influence cognitive alterations in the elderly population with age-related brain pathologies, and specific dietary interventions might, therefore, confer preservatory protection to neural structures. Although Mediterranean and ketogenic diets have been studied, no clear guidelines have been implemented for the prevention or treatment of ND in clinical practice. Murine models have shown that intermittent fasting and caloric restriction (CR) can counteract disease processes in various age-related disorders, including NDs. The objective of this perspective is to provide a comprehensive, comparative overview of the available primary intervention studies on fasting and CR in humans with ND and to elucidate possible links between the mechanisms underlying the effects of fasting, CR, and the neuropathology of ND. We also included all currently available studies in older adults (with and without mild cognitive impairment) in which the primary endpoint was cognitive function to provide further insights into the feasibility and outcomes of such interventions. Overall, we conclude that nutritional intervention trials focusing on fasting and CR in humans with ND have been neglected, and more high-quality studies, including longitudinal clinical intervention trials, are urgently needed to elucidate the underlying immune–metabolic mechanisms in diet and ND.

**Statement of Significance**

This perspective provides a pioneering synthesis of clinical intervention trials examining the effects of fasting and caloric restriction on individuals suffering from neurodegenerative diseases, marking a comprehensive analysis on this topic.

## Introduction

Fasting (i.e., caloric restriction [CR] in various forms) has been used as an intervention to promote health since the beginning of civilization and has spread independently among different regions, cultures, and religions worldwide [1]. It is believed to have already been established as a treatment method by Hippocrates in the 5th century BCE and has been used ever since by numerous medical schools to treat acute and chronic diseases [2]. Various practices of CR using fasting have repeatedly shown remarkable health benefits [3,4].

Neurodegenerative diseases (NDs) comprise a range of complex medical conditions that affect neurons in the brain and possibly extend to the spinal cord and the peripheral nervous system [5]. The most common such diseases are Alzheimer’s disease (AD), Parkinson’s disease (PD), and multiple sclerosis (MS) [6]. NDs, especially AD, have been on the rise in recent decades, mostly because of aging populations [7]. Although recent studies on several different treatment approaches, such as monoclonal antibodies against plaques, stem cell therapy, or nanotherapeutics, present promising treatment approaches, no cure for these diseases currently exists [[7], [8], [9], [10]].

Genetic factors and age are key players in the onset and development of NDs; however, environmental and lifestyle factors also play an important role in their development [11]. In addition to physical activity and cognitive exercise, nutrition has emerged as a major factor influencing ND pathology [12]. The Mediterranean diet (MD) and ketogenic diet (KD) have been associated with neuroprotective effects in ND, based on the inhibition of glycolysis, improved mitochondrial respiration, decreased production of reactive oxygen species, and prevention of neuronal apoptosis [13].

Although the beneficial effects of fasting have been observed in a wide variety of diseases, such as rheumatoid arthritis, or even during chemotherapy, a possible association with preventing or treating ND is still unclear [14,15]. Although a large body of work exists in animal models, including models for AD, PD, and stroke, showing that intermittent fasting and CR have beneficial effects on health and can counteract disease processes, few human studies have been conducted to date [16].

**FIGURE 1:**
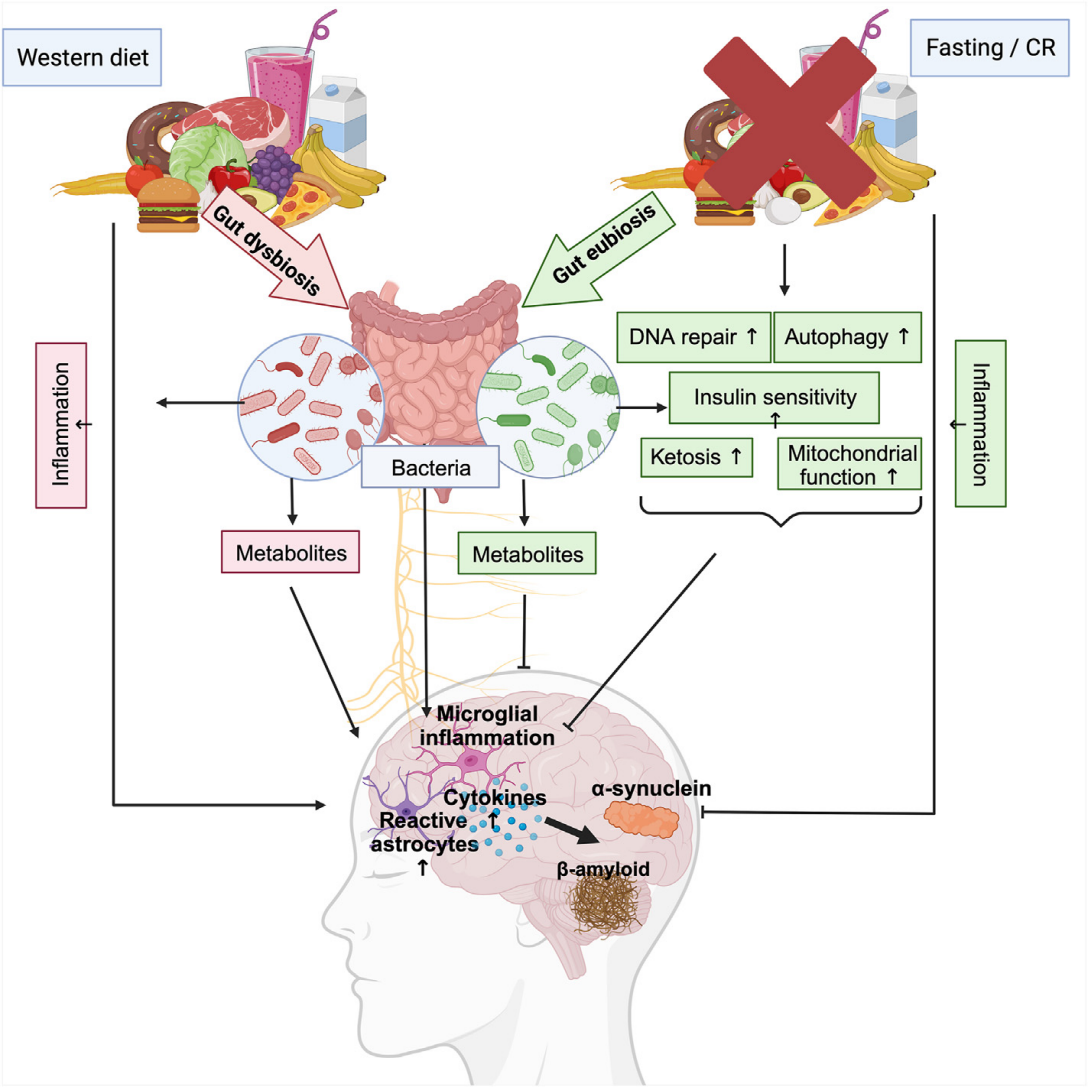
Underlying mechanisms. This figure illustrates the possible underlying mechanisms of the beneficial impact of fasting and CR on ND. While a typical western diet usually leads to gut dysbiosis and an increase in inflammation, the abstinence or reduction of food can have a positive impact on cognitive function by changing the gut microbiota composition and its metabolite secretion [41,42,46]. Several additional mechanisms including DNA repair, increased autophagy, upregulated mitochondrial function, increased insulin sensitivity, increased ketone body production, and decreased overall inflammation may result in beneficial impacts on cognitive function [[73], [74], [75]]. Abbreviations: CR, calorie restriction; ND, neurodegenerative disease. This image was generated using BioRender software (www.biorender.com).

Here, we provide a comprehensive, comparative overview of the available data from primary intervention studies on fasting in humans with NDs, including all currently available studies in older adults (with and without mild cognitive impairment [MCI]) in which the primary endpoint was cognitive function, to provide further insights into the feasibility and outcomes of such interventions. Furthermore, we elucidate the possible links between the mechanisms underlying the effects of fasting and the neuropathology of ND (Figure 1).

## Current Status of Knowledge

### Fasting and caloric restriction

Fasting is defined as the voluntary abstinence from caloric ingestion for a limited time period. There are various forms of fasting and CR. Prolonged fasting (PF) typically lasts between 48 h and 21 d, with an intake of less than 350 kcal/d [17]. Intermittent fasting (IF) is an umbrella term that covers different approaches, including alternate day fasting, where complete or severe energy restriction occurs every other day, and time-restricted eating (TRE), where food intake is restricted to a specific time period each day. The most popular forms of IF are the TRE 16:8 method, with food intake ad libitum for 8 h followed by 16 h of fasting, and the 5:2 diet where calorie restriction (∼600 kcal) occurs on 2 consecutive or nonconsecutive days per week. The latter is sometimes referred to as periodic fasting. CR is defined as a reduction in energy intake by at least 20% to 30% for 3 mo or more with an appropriate nutritional composition [18]. Finally, the fasting-mimicking diet (FMD), a combination of CR and IF, is becoming more popular. FMD is a low-calorie, low-protein, high unsaturated fatty acid (FA) diet characterized by periodic cycles of 3 to 7 d on a very low-calorie diet (∼800–1100 kcal), providing essential macro- and micronutrients [19,20].

### Alzheimer’s disease

AD is the most prevalent ND and most common cause of dementia. More than 50 million patients are affected worldwide, and the numbers have increased dramatically over the past decades due to demographic changes in the global population, with people aged 65 and above outnumbering children under the age of 5 for the first time in 2018 [21,22]. AD is characterized by cognitive dysfunction, memory loss, and abnormal personality [22]. β-amyloid protein, the main component of senile plaques, and neurofibrillary tangles, composed of hyperphosphorylated tau protein, are considered key factors in the propagation of AD pathology [23]. Mendelian randomization studies provide evidence of a causal association between glycemic traits and AD [24]. Impaired glucose metabolism may be intrinsic to AD pathogenesis, contributing to oxidative damage, inflammation, and reduced energy metabolism [25].

A multitude of studies of dietary interventions and supplements has been conducted in humans with AD [26]. Medium chain triglyceride supplementation has shown promising effects in glucose hypometabolism in AD by increasing ketone levels and providing an alternative energy source to the brain [27,28].

However, to date, only one fasting study has been conducted on humans with mild AD or MCI. This randomized, placebo-controlled, 1-y study is ongoing and compares monthly, 5-d FMD cycles to a placebo diet where one meal is replaced with a pasta or rice-based meal 5 d per month. Twenty-eight patients have been enrolled to date (range 55–80 y), with the aim of enrolling 40 patients. The FMD group took a variety of dietary supplements for 25 d between the FMD cycles noted for fasting-mimicking and neuroprotective properties, whereas the placebo group did not. Initial data, 6 mo into the study, reported that 5 patients dropped out of each group for a variety of reasons, including poor acceptance and worsening nutritional status. FMD-emergent adverse events were mild to moderate. Diet compliance was good, and the authors considered 5-d FMD cycles administered once per month feasible and safe for patients with early AD or MCI. Information regarding potential cognitive changes will be reported at the end of the study [20].

### Mild cognitive impairment

MCI is a transitional stage between the expected cognitive decline that occurs with age and the more serious decline observed in AD. Estimates of the etiology of AD dementia among patients with MCI range from 40% to 75% in different populations. The estimates include both those where MCI diagnosis was made clinically and in combination with biomarkers [29]. One interventional fasting trial and one CR trial have been conducted on humans with MCI.

Horie et al. [30] reported on a single-center, prospective controlled trial in obese patients suffering from MCI, aged 60 y or older. Patients were randomly allocated to conventional medical care alone (*n* = 40) or together with nutritional counseling (*n* = 40), aiming to promote weight loss through CR (a recommended calorie deficit of approximately 500 kcal/d for 12 mo). Significant weight loss was observed in all 75 patients completing the study, and cognitive test results improved without a difference between the groups. In the analysis, a decrease in BMI was associated with improvements in cognitive tests. Thus, the authors concluded that intentional weight loss was associated with improved cognition in MCI patients [30].

Ooi et al. [31] prospectively studied 99 elderly subjects with MCI of Malay ethnicity for 36 mo. The participants were grouped according to whether they regularly practiced IF (r-IF, *n* = 37), irregularly practiced IF (i-IF, *n* = 35), or nonfasters (n-IF, *n* = 27). IF was practiced by fasting on Mondays and Thursdays every week (Sunnah fasting) beginning from sunrise to sunset. Drinking was not permitted during fasting. After 36 mo, more MCI subjects in the r-IF group reverted to no cognitive impairment and lack of disease (categorized as successful aging) (24.3%) than those in the i-IF (14.2%) and n-IF groups (3.7%). The r-IF group showed a significant increase in the oxidative stress markers superoxide dismutase and malondialdehyde, activity and reduction in body weight, levels of insulin, fasting blood glucose, the inflammatory marker C-reactive protein, and DNA damage. Furthermore, metabolomics analysis showed that IF may modulate cognitive function via various metabolite pathways [31].

### Parkinson’s disease

PD is an age-related ND that affects 0.4% to 2% of the population over 65 y worldwide and is the second most common progressive neurodegenerative disease, with men being 1.5 times more likely to be affected than women [32,33]. Cardinal symptoms include motor deficiencies, such as tremors, bradykinesia, and rigidity, but also include a wide range of nonmotor symptoms, such as hyposmia, depression, insomnia, or cognitive impairment, constipation, and rapid eye movement sleep behavior disorders, severely impacting patients’ quality of life [[33], [34], [35], [36]]. The main neuropathological manifestations include neuroinflammation, degeneration of dopaminergic neurons, and accumulation of α-synuclein, a major component of Lewy bodies, in the dopaminergic substantia nigra [37,38]. The loss of dopaminergic neurons in PD involves mechanisms of inflammatory and autoimmune responses, with microglial activity being the major driver [39].

It is well established in the PD community that diet has a major impact on the disease. The 2 different dietary approaches shown to have beneficial effects on the outcome of PD are the MD and KD [40]. This is surprising, as these diets vary significantly in their composition. Whereas the MD is rich in antioxidants and fibers from fruits and vegetables, nuts, white grains, and healthy fats, the KD is usually high in saturated fats of animal origin and low in carbohydrates and fibers. These opposing dietary patterns imply the action of complex underlying mechanisms beyond the simple macro- and micronutrient composition. As both a high fiber intake and a metabolic switch have major impacts on the gut microbiome, the microbiota–gut–brain axis could be a key factor in modulating the onset and disease course of PD [41,42]. As fasting is also known to have a major impact on the composition of the gut microbiome, previous findings may indicate a beneficial effect of PF and/or IF on PD. However, no primary human studies have investigated fasting or CR in PD patients to date. A currently ongoing clinical trial, the ExpoBiome study, is investigating PF for the first time in patients with PD [43].

### Multiple sclerosis

MS is a disease of the central nervous system, and while it is generally characterized as an autoimmune disorder, it is characterized by demyelination and neurodegeneration mediated by both T and B cells. MS is considered the leading cause of nontraumatic neurological disability in young adults. It is a heterogeneous disease in which most patients suffer from a relapsing form where discrete episodes of illness are followed by possible complete or partial remission, but 10% experience progression from the outset [44]. Metabolic syndrome and other closely related disorders such as diabetes and hyperlipidemia are overrepresented in patients with MS and are strongly associated with adverse outcomes [45]. Therefore, it is possible that CR and fasting may be important interventions impacting the development of disease [46].

Choi et al. [47] studied the effects of a low-calorie, low-protein FMD on patients with relapsing–remitting MS. A total of 60 patients were randomly assigned to a control diet (*n* = 20), the KD for 6 mo (*n* = 20), or a single cycle of modified human FMD for 7 d (*n* = 20) followed by the MD for 6 mo. Health-related quality of life and mental health were assessed at baseline, month 3, and month 6, and both the KD and FMD cohorts displayed meaningful to statistically significant improvements in all areas. The interventions were well-tolerated, and there were high compliance rates. However, adverse events were reported in all 3 groups, with airway infections (adverse events) and lower urinary tract infections (serious adverse events) being the most common [47].

In a more recent but smaller study, Fitzgerald et al. [48] randomly allocated 36 patients with MS to 3 diets for 8 wk: daily CR diet (22% daily reduction in energy needs), intermittent CR diet (75% reduction in energy needs, 2 d/wk; 0% reduction, 5 d/wk), or a weight-stable diet (0% reduction in energy needs, 7 d/wk). Adherence to daily CR was better than that to intermittent CR, with 86% completing the trial overall. Both CR diets were associated with significant improvements in emotional well-being and depression scores compared with the control weight-stable diet. No significant adverse effects were observed [48].

Bock et al. [49] studied a cohort of 60 relapsing–remitting MS subjects who were randomly allocated to a control diet (*n* = 9), a calorie restricted diet (single cycle of 7-d CR with 200–350 kcal/d was performed at study outset; afterwards a 3-d stepwise reintroduction to an isocaloric common diet, *n* = 14) or an adapted KD (average daily intake of <50 g carbohydrates, >160 g fat, and ≤100 g protein intake per day for 6 mo, n = 17). Serum neurofilament light chain (sNfL) measurements were performed at baseline, 3 mo, and 6 mo. sNfL levels are emerging biomarkers for neuroaxonal damage, and elevated sNfL levels are indicative of axonal injury [50]. An unexplained statistically significant increase in sNfL occurred at 3 mo in all 3 groups in an intragroup comparison. Only participants consuming the adapted KD showed a statistically significant decrease from baseline to 6 mo compared to the control group at the same time point.

The most recent study examined 10 relapsing–remitting MS patients that were randomized to an intermittent calorie restriction (*n* = 5) or control group (*n* = 5) for 12 wk. IF was defined as a reduction in daily calorie intake to ∼25% of the usual intake on 2 nonconsecutive days per week. Significant improvements were observed in cortical volume and thickness, and neuroinflammation was mitigated [51].

### Fasting and cognition in older adults

With age, many biological changes contribute to a progressive decline in physical function and cognition. Several contributing factors appear to accelerate this process, including low activity levels, excessive calorie intake, and body fat. Numerous studies in humans and animal models have shown that fasting has both beneficial and negative effects on cognition. Most human studies, however, have been performed in younger adults, with only a small number having been conducted in older adults.

In the oldest study by Witte et al. [52], conducted in 2009, 50 healthy, normal to overweight elderly subjects (mean age 60.5 y) were stratified into 3 groups: CR (30% reduction, *n* = 20), relative increased intake of unsaturated FAs (20% increase, unchanged total fat, *n* = 20), and control (*n* = 10). Memory performance was assessed under standardized conditions at baseline and after 3 mo of intervention. A significant increase in verbal memory scores after CR was observed, which was most pronounced in those with the best adherence to the diet. No significant memory changes were observed in the other groups [52].

Siervo et al. [53] recruited both middle-aged and older obese individuals. In the older age group (*n* = 26; mean age = 64.5 y), 12 individuals completed the study. Energy intake was reduced by 40% relative to an individual’s calculated resting energy expenditure, and the weight loss target for each subject was 8% to 12% of the initial body weight. The duration to achieve this weight loss was 116.6 ± 27 d. Global cognitive performance, as measured by the Mini-Mental State Examination, only improved significantly in older individuals, whereas both age groups showed a significant improvement in the Trail-Making Test B, which measures visual search, scanning, processing speed, mental flexibility, and executive functions.

TRE was evaluated in a small group of 10 overweight adults (≥65 y) at risk for or with mobility impairment. The intervention was a TRE dietary pattern following the 16:8 method, which lasted for 4 wk. While compliance was high and significant mean weight loss was observed, no change in cognitive function was measured using the Montreal Cognitive Assessment 30-point questionnaire for MCI. Few adverse events were reported (e.g., headache and dizziness) [54].

Hugenschmidt et al. [55] studied sedentary, obese adults (65–79 y) with normal cognition in a randomized trial comparing 3 groups: 20-wk aerobic exercise program only (*n* = 28), moderate (∼250 kcal) CR with the exercise program (*n* = 30), or high (∼600 kcal) CR with the exercise program (*n* = 30). The participants were evaluated at multiple time points for cognitive outcomes using a cognitive assessment battery. Randomization to CR did not significantly alter overall cognitive function compared to aerobic exercise alone, nor were there between-group differences in any individual executive function test up to 24 mo postrandomization. Compliance and adherence were excellent, and none of the participants dropped out because of an intervention-related adverse event.

A relatively large group of 107 elderly, obese individuals was randomly allocated to 4 groups: CR (500–750 kcal/d less than daily requirement, *n* = 26), CR plus exercise (*n* = 28), exercise only (*n* = 26), or control (*n* = 27). The goal was to achieve 10% weight loss in the first 6 mo, followed by weight maintenance for another 6 mo. Compliance was >82% in all 4 groups, and no adverse effects were noted. In the overall sample, cognitive function improved, but randomization to CR did not significantly change executive function compared to exercise alone. Furthermore, there were no between-group differences in any individual executive function test following the intervention or at long-term follow-up. Adding CR to exercise was associated with a modest improvement in the Mini-Mental State Examination score [56].

A small group of 11 sedentary, overweight, or obese older women (63–80 y) was randomly allocated to a 48-h zero-calorie diet with water provided ad libitum or their usual diet. A paired crossover design was used, with the interventions being at least 2 wk apart. Before-diet measurements were taken 1 d before the intervention, and after-diet measurements were taken immediately after the acute fasting ended. Cognitive performance was assessed using a test battery. The zero-calorie diet significantly prolonged the reaction time in a 2-choice reaction time test. Other cognitive tests were unaffected [57].

The largest study we identified included 185 obese, elderly individuals who were randomized to the MD plus CR lifestyle intervention (∼25% CR to achieve ∼5–7% weight loss, *n* = 75), MD lifestyle intervention only (*n* = 73), or their usual diet (*n* = 37). Participants were followed up for 14 mo, with the main measurements presented in the paper taken at baseline and 8 mo (completion of the active intervention phase). Although the CR group lost significant weight, the MD lifestyle intervention with and without CR did not significantly affect cognitive function compared with controls [58].

### Parallels of fasting and ketogenic diet

To better understand the mechanisms of fasting and CR in humans with ND, it is worthwhile to examine several interventional KD studies conducted in patients with PD and AD. Both the effects of fasting and KD are thought to at least partly be based on the switch from glucose to fat metabolism and on a consequent change in the gut microbiome composition. KD is a low-carbohydrate, high-fat diet that induces a state of ketosis. Possible neuroprotective effects of KD through enhanced mitochondrial function, reduced inflammation, improved energy metabolism, and increased production of ketone bodies have been suggested [59].

The first trial of KD in patients with PD was published in 2005 by Vanitallie et al. [60]. Although improvements in Unified Parkinson’s Disease Rating Scale scores could be seen, one must note that the sample size was small (*n* = 5) and the duration of diet implementation was only 28 d [60]. In 2021, Krikorian et al. [61] compared high-carbohydrate and very low-carbohydrate (VLC) diets in 23 older adults with MCI; after the 6-wk intervention period, significant cognitive improvement was observed in the VLC group. A significant improvement in the Movement Disorder Society-Sponsored Revision of the Unified Parkinson’s Disease Rating Scale (MDS-UPDRS) scores was observed in both arms of an 8-wk interventional trial by Phillips et al. [62], using low-fat diet compared to isolcaloric KD in 47 patients; the ketogenic group showed a significantly higher improvement for MDS-UPDRS Part 1 with a total reduction of 41% from baseline scores [62]. Koyuncu et al. [63] found a significant improvement in voice quality in patients with PD after consuming a KD for 3 mo.

Few studies have been conducted on KD in AD. Brandt et al. [64] found a significant cognitive improvement after using a modified Atkins diet for 6 wk. However, of the initially 27 enrolled patients, only 9 diet patients and 5 control patients completed the study [64]. The trial of Phillips et al. [62] reported in 2021 on the KD in patients with AD noted better daily function and quality of life, likely due to increased synaptic plasticity and reduced inflammation [52].

Ketogenesis creates ketone bodies like acetoacetate and β-hydroxybutyrate, serving as energy sources and offering antioxidative benefits that support mitochondrial function and reduce inflammation in Alzheimer's ([65], [66], [67]). Additionally, the microbiota–gut–brain axis is increasingly recognized in NDs where gut health has been linked to PD development [68]. Diet influences gut health significantly [33]. However, sustaining KD poses risks like malnutrition cardiovascular diseases, particularly in the elderly [69].

## Conclusion

Of the 14 studies reviewed for this comprehensive perspective, 4 studies included patients with MS and 1 study included patients with AD. No fasting or CR studies were identified for PD patients, except for the ongoing ExpoBiome study [43], and all further studies included subjects with MCI or elderly normal weight, overweight, or obese subjects (Table 1). Studies in subjects with ND or MCI generally show positive effects on disease symptoms and/or cognition. However, the impact of fasting or CR on cognition in older adults produced heterogeneous results. The overall number of studies in humans is low. Other limitations include small sample sizes, short duration of the intervention, timing of cognitive measurements, or complex study designs that included exercise. Several studies focused on weight loss as the primary outcome, which can have a beneficial impact on cognitive function; however, the magnitude of weight loss did not correlate with the magnitude of cognitive improvement [70,71].

**TABLE 1:** Interventional studies in individuals with NDs or the elderly (with cognitive function as endpoint)

Reference	Disease or condition	Main endpoint(s)	Diet(s)	Total *n*	Age (y)	% Female
Anton (2019) [54]	Elderly, obese	Cognitive function	TRE 16:8	10	M = 77.1	60
Bock (2022) [49]	Multiple sclerosis	sNfL levels	Fasting, KD, or control	60	M = 43.1, 45.7, 50.0	67, 76, 86
Choi (2016) [47]	Multiple sclerosis	Quality of life, mental health	FMD, KD, or control	60	M = 44.8	79
Fitzgerald (2018) [48]	Multiple sclerosis	Change in emotional well-being, depression	Intermittent CR, daily CR, or control	36	M = 37.4	81
Horie (2016) [30]	MCI, overweight	Cognitive function	CR or control	80	M = 68.1	83.7
Hugenschmidt (2019) [55]	Elderly, obese	Cognitive function	High CR plus exercise, medium CR plus exercise, or exercise only	88	M = 69.0	68
Napoli (2014) [56]	Elderly, obese	Cognitive function, quality of life	CR, CR plus exercise, exercise only, or control	107	M = 70, 70, 70, 69	65, 57, 61, 67
Ooi (2020) [31]	MCI	Cognitive function	Regular IF, irregular IF, or control	99	M = 68.7, 67.9, 69.1	37.8, 57.1, 44.4
Rahmani (2023) [51]	Multiple sclerosis	Cortical thickness, volume, perfusion, neuroinflammation	CR or control	10	M = 46	80
Rangan (2022) [20] 1	AD or MCI	[Cognitive function]^1^	FMD or control	28^1^	M = 71^1^	46^1^
Siervo (2012) [53]	Elderly, obese	Cognitive function	CR	26	M = 64.5	88
Solianik (2020) [57]	Elderly, overweight to obese	Cognitive function	PF, paired crossover design	11	Range = 63–80	100
Tussing-Humphreys (2022) [58]	Elderly, obese	Cognitive function	CR with MD, MD, or control	185	M = 66.3	85.9
Witte (2009) [52]	Elderly, normal to overweight	Cognitive function	CR, unsaturated FA enhancement, or control	50	M = 60.5	58

Abbreviations: AD, Alzheimer’s disease; CR, caloric restriction; FMD, fasting-mimicking diet; IF, intermittent fasting; KD, ketogenic diet; MCI, mild cognitive impairment; MD, Mediterranean diet; ND, neurodegenerative disease; PF, prolonged fasting; sNfL, serum neurofilament light chain; TRE, time-restricted eating.

1 Study recruitment is ongoing.

Possible underlying mechanisms based on the metabolic switch have been elucidated in the section *Parallels of fasting and ketogenic diet*, as well as the risks and difficulties of the latter. PF or TRE can bypass the challenges encountered in a KD, as fasting is temporary, and a balanced diet can be reinitiated after the respective fasting periods. In addition, PF and TRE have been associated with positive outcomes in several conditions and diseases such as obesity, type 2 diabetes, and rheumatoid arthritis [14,72]. Patients with ND following a TRE pattern or PF could experience benefits beyond cognitive improvement [[73], [74], [75]].

Dietary recommendations for ND and the imminent application of the latter as a standard therapeutic intervention in daily clinical practice are of critical importance. Ongoing clinical trials, such as the ExpoBiome study [43], will provide insight into the mechanisms of fasting and the microbiota–gut–brain axis in relation to ND. Overall, this perspective emphasizes the need for additional clinical trials studying various fasting protocols, as this might potentially constitute a powerful new tool for preventing and treating ND.

## Author contributions

The authors’ responsibilities were as follows—BH, KR, JGS: conceptualized the research approach, planned and drafted the manuscript outline; BH, KR: wrote the paper; HE: contributed to literature research; JGS: reviewed and edited the manuscript; and all authors: read and approved the final manuscript.

## Funding

This research was funded in part by the Luxembourg Centre for Systems Biomedicine (LCSB) and by the Luxembourg National Research Fund (FNR), grant reference PRIDE/11823097. For the purpose of open access, and in fulfillment of the obligations arising from the grant agreement, the author has applied a Creative Commons Attribution 4.0 International (CC BY 4.0) license to any Author Accepted Manuscript version arising from this submission.

## Conflict of interest

The authors report no conflicts of interest.
